# Hedgehog on the Move: Glypican-Regulated Transport and Gradient Formation in *Drosophila*

**DOI:** 10.3390/cells13050418

**Published:** 2024-02-27

**Authors:** Carlos Jiménez-Jiménez, Kay Grobe, Isabel Guerrero

**Affiliations:** 1 Centro de Biología Molecular Severo Ochoa (CSIC-UAM), Universidad Autónoma de Madrid, Nicolás Cabrera 1, E-28049 Madrid, Spain; carjim@cbm.csic.es; 2 Institute of Physiological Chemistry and Pathobiochemistry, University of Münster, Waldeyerstrasse 15, 48149 Münster, Germany

**Keywords:** glypicans, heparan sulphate proteoglycans, Dally, Dally like, Hedgehog

## Abstract

Glypicans (Glps) are a family of heparan sulphate proteoglycans that are attached to the outer plasma membrane leaflet of the producing cell by a glycosylphosphatidylinositol anchor. Glps are involved in the regulation of many signalling pathways, including those that regulate the activities of Wnts, Hedgehog (Hh), Fibroblast Growth Factors (FGFs), and Bone Morphogenetic Proteins (BMPs), among others. In the Hh-signalling pathway, Glps have been shown to be essential for ligand transport and the formation of Hh gradients over long distances, for the maintenance of Hh levels in the extracellular matrix, and for unimpaired ligand reception in distant recipient cells. Recently, two mechanistic models have been proposed to explain how Hh can form the signalling gradient and how Glps may contribute to it. In this review, we describe the structure, biochemistry, and metabolism of Glps and their interactions with different components of the Hh-signalling pathway that are important for the release, transport, and reception of Hh.

## 1. Introduction

Communication between cells is essential for the proper development of all multicellular organisms. Cellular communication is often mediated by the activity of specific signalling molecules called morphogens. During embryogenesis, morphogens are defined as being produced at a localised source and acting at significant distances from the source. As they spread, morphogens are thought to be progressively distributed within a morphogenetic field, which is defined as the area in which recipient cells respond by activating different target genes depending on the level of signal [1]. Therefore, the release of morphogens from producing cells, their graded distribution across the morphogenetic field, and the ability of recipient cells to respond specifically to different ligand concentrations must be tightly regulated to ensure proper tissue formation and function.

Both the graded distribution and receptor-mediated internalisation of morphogens require a family of cell surface-associated heparan sulphate proteoglycans (HSPGs), the glypicans (Glps). Glps have evolved as essential modulators of key regulatory proteins such as Wnts, Hedgehog (Hh), Fibroblast Growth Factor (FGF), Bone Morphogenetic Proteins (BMPs), and the Jak/Stat signalling pathway by acting on signal spreading and receptor activation, which in turn controls signal transduction and fate in target cells. Glps can also act as co-receptors for morphogens, enhancing or inhibiting their binding to primary cell surface receptors [2]. In addition, Glps can regulate the intracellular trafficking and degradation of morphogens, affecting their availability and activity [3,4]. Consistent with the importance of these activities in influencing critical signalling pathways, dysregulated Glp function has also been implicated in various diseases such as cancer, inflammation, and neurodegeneration [5].

One of the best-studied experimental systems for testing Glp function is the development of the *Drosophila melanogaster* wing imaginal disc, which gives rise to the adult wing. It is well established that wing development is strongly dependent on unimpaired Hh morphogen production, release, and extracellular spreading. Hh is also known to be involved in stem cell maintenance, axon guidance, cell migration, and oncogenesis in a wide range of organisms [6]. In the developing wing, the production, transport, release, and reception of Hh must therefore be kept under tight spatial and temporal control in order for Hh to fulfil its signalling function. An important feature of all invertebrate and vertebrate Hh family members is their post-translational modification by a covalently C-terminally attached cholesterol [7] and an N-terminally attached palmitic acid during biosynthesis [8]. Both lipids promote a tight association of Hh with the outer cell membrane leaflet, raising the important question of how the dual-lipidated morphogen is released from the plasma membrane and delivered to recipient cells in a robust, yet scalable manner. Over the past two decades, several modes have been proposed to overcome the apparent paradox that a tightly membrane-associated protein can signal to recipient cells over considerable distances: (1) the release of lipidated Hh by micelle formation [9]; (2) association of lipidated Hh with lipoprotein particles (LPP) [10,11]; (3) association of lipidated Hh with exosomes [12,13]; (4) Hh association with a soluble factor called Shifted (Shf) [14,15]; (5) proteolytic processing to release the Hh-signalling domain from both lipidated membrane anchors [16]. All of these modes are expected to convert insoluble Hh into protein complexes that can be transported by diffusion. Another proposed mechanism to regulate Hh distribution is by specialised filopodia (called cytonemes) that transport ligands to receptors on the surface of the signal-receiving cell while still attached to the plasma membrane of the signal-generating cell [17]. It is conceivable that these different modes of Hh release and trafficking operate in different tissues and developmental contexts or may act together in the same tissue to fine-tune Hh biofunction. Given the essential role that Glp expression plays in Hh signalling, all proposed mechanisms of Hh release and relay are expected to depend on Glp expression in the morphogenetic field and/or on producing and receiving cells.

In this review, we discuss the role of Glps in Hh signalling and gradient formation, taking into account the most recent findings on Glp-HSPG interactions with the Hh ligand or with other components of the Hh signalling pathway. We also discuss two new hypotheses on how Glps might support the two models of Hh gradient formation. Finally, given that the roles that Glps play during development and disease are related to their influence on critical signalling pathways, a better understanding of how Glps modulate morphogen signalling is crucial for advancing our knowledge of HSPG functions in biology and medicine.

## 2. Structure, Biochemistry and Metabolism of Glp-HSPGs in the Extracellular Matrix 

**Figure 1:**
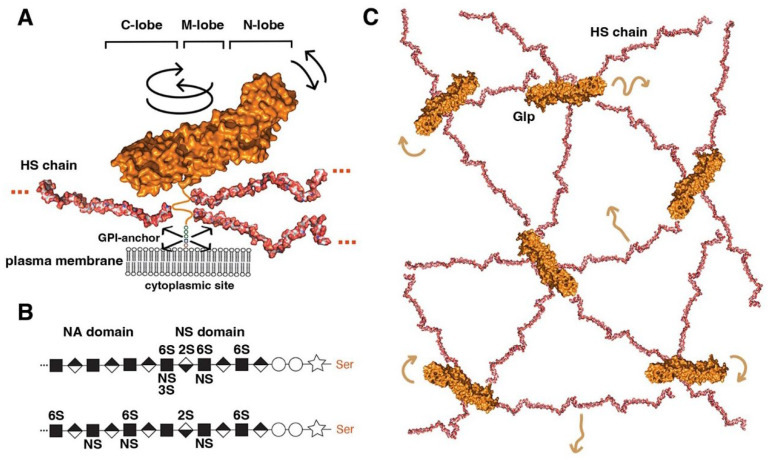
Overview of Glp structure and predicted cell surface distribution. (**A**) Representation of Glp1 lacking the most C-terminal disordered domain (Glp1DC) [24]. The disordered C-terminal Glp domain (aa Asn474-Ser530) contains the attachment sites of three closely spaced HS chains located close to the folded core (linked to serine residues Ser486, Ser488, and Ser490) and connects the Glp core domain to the GPI anchor. The HS structures were resolved separately [25] and manually added to the schematic. Three different lobes can be assigned to the Glp1DC structure: The cysteine-rich N-lobe, the central or M-lobe, and the C-lobe (also called the protease lobe because it has been described in many Glp family members to be susceptible to processing by furin proteases [26]). The structure of Glp1 is very similar to that of *Drosophila melanogaster* Dlp, despite only 25% sequence similarity. Note that the GPI membrane anchor and the unstructured flexible C-terminal domain give the core a large degree of freedom to tilt, move laterally, and rotate relative to the membrane (arrows). Shown are protein data bank (pdb) structures 3irl (HS) and 4ad7 (Glp). The structures are not to scale. (**B**) HS biosynthesis starts with a xylose residue (star) linked to a serine of the proteoglycan protein, followed by two galactose (circles) and a glucuronic acid residue (diamond). The subsequent addition of an N-acetylglucosamine residue (square) to the tetrasaccharide linker region initiates the biosynthesis of HS chains by the HS copolymerase complex. The growing chain (eventually consisting of 50–150 sugar residues) is simultaneously modified by N- and 2O-, 3O-, and 6O-sulphotransferases and an epimerase that generates iduronic acid residues (inverted diamond) from glucuronic acid residues. In vertebrates, high sulphated domains (NS domains) are separated by low-sulphated domains called NA domains (top). In contrast, *Drosophila* HS consists of a continuous sulphated domain (bottom) [27]. (**C**) A bird’s eye view of modelled multiple highly dynamic (brown arrows) interaction sites of Glp HS chains with neighbouring Glp core proteins, other HS chains and lipid head groups at the cell surface [28].

A general function of the extracellular matrix is to modulate the activities of soluble growth factors and morphogens by sequestration, stabilisation, facilitation or inhibition of transport, and receptor binding. In mammals, cell surface-associated proteins that fulfil these roles include CD44, NG2, neuropilin-1, and the syndecans. Another group of extracellular matrix proteins known to fulfil all these functions for multiple soluble ligands in a context-dependent manner is the Glp protein family. This family has been conserved during animal evolution in both invertebrates and vertebrates [18,19], with six Glps (Glp1 to Glp6) identified in mammals [20] and two (Division abnormally delayed (Dally) and Dally like protein (Dlp)) in *Drosophila melanogaster*. On the basis of amino acid homology, mammalian Glps can be divided into two distinct groups. The first group includes Glp1, Glp2, Glp4, and Glp6 with 35–63% sequence similarity; the second group includes Glp3 and Glp5, with 54% sequence similarity [19,21], whereas the homology between the two groups is only 17–25%. The two *Drosophila* Glps Dally and Dlp [22] are representatives of each group: Dally is an ortholog of mammalian Glp3 and 5, and Dlp is an ortholog of Glps1, 2, 4 and 6 [19]. Crystallography and structural analysis support this relationship, revealing similar elongated, alpha-helical folds for Dlp and Glp1, despite the fact that these two Glps share only 25% sequence homology [23]. Furthermore, both structures do not appear to be homologous to any other known protein structure, suggesting unique functional roles for vertebrate and invertebrate Glp core proteins [23]. Glp core proteins are ~60 to 70 kDa in size and share three common structural features (Figure 1A). 

The first structural feature is a globular cysteine-rich N-lobe, which is similar to the cysteine-rich domains found in the Wnt receptor Frizzled and in the Hh-signalling transducer Smoothened [29]. The tertiary structure of the Glp N-lobe is likely to be constant among family members, due to the presence of 14 highly conserved cysteine residues that form stabilising disulphide bonds (Figure 1A). The second and third structural features of Glps are a central (or M) lobe and a C-terminal lobe susceptible to furin processing [26]. The fourth structural feature of all Glps is a disordered linker domain at the C-terminal end that connects the core protein to a glycosylphosphatidylinositol (GPI) anchor [30] for Glp insertion into the outer leaflet of the cell membrane (Figure 1A). This disordered linker, containing more than 50 amino acids, allows Glps to rotate freely and move laterally at the cell surface. The lack of a cytoplasmic domain prevents Glp internalisation by caveolin- and clathrin-coated vesicles. Instead, the GPI-anchor can target the molecule to recycling endosomes in a cdc42-dependent manner in mammals [31]. 

The fifth structural feature is a short peptide adjacent to the linker region that is decorated with varying numbers (2 to 5) of heparan sulphate (HS) glycosaminoglycan chains (Figure 1A). These chains on the Glp core protein are produced by most vertebrate and invertebrate cell types [32,33,34,35] (Figure 1B). Heparan sulphate (HS) biosynthesis starts with the addition of a tetrasaccharide linker to dedicated serine residues of the core protein in the Golgi compartment, followed by the synthesis of a linear carbohydrate backbone consisting of alternating glucuronic acid or iduronic acid/N-acetylglucosamine disaccharide units by enzymes called the exostosins (Exts) [36]. In *Drosophila*, the formation of HS glycosaminoglycan chains is catalysed by glycosyltransferases encoded by members of the EXT gene family: *tout-velu (ttv)* [37], *brother of tout-velou (botv)*, and *sister of tout-velou (sotv)* [38]. The nascent chains are then modified by one or more of the four N-deacetylase/N-sulphotransferase (Ndst) isoforms identified in vertebrates [39] (called *sulfateless* (*sfl*) in *Drosophila*) and further modified by sulphotransferases and a GlcA-C5 epimerase [40]. The resulting HS chains vary in size from ∼5 to 70 kDa (corresponding to chain lengths ranging from 40 nm to more than 160 nm [41]) and are located within 50 amino acid residues of the membrane anchor. As a result, HS chains are positioned close to the cell membrane, allowing them not only to bind many soluble growth factors, chemokines, cytokines, and morphogens via their strong negative charge to bring them closer to the cell surface but also to bind their receptors and polar lipid head groups on the cell surface [42,43]. All-atom molecular modelling and simulation of GPI-anchored Glp1 with three HS chains in a lipid bilayer to explore their possible dynamics and interactions suggested multi-site interactions between Glps and the plasma membrane (Figure 1C) [28]. These dynamic interactions are facilitated by the unstructured C-terminal Glp domain linking the core domains to the GPI anchor, which gives the molecule a large degree of freedom to tilt, rotate, and move laterally at the cell membrane. In particular, the highly dynamic and flexible HS chains can make contact with neighbouring Glp1 protein cores, with other HS chains in the vicinity and surrounding head groups (Figure 1C). These simulations make it possible to imagine Glp HS chains forming a highly dynamic, negatively charged network at the cell surface for efficient interaction with growth factor/morphogen ligands and with their receptors.

## 3. Glp Expression Patterns and Their Influence on Morphogenetic Signalling in *Drosophila*

**Figure 2:**
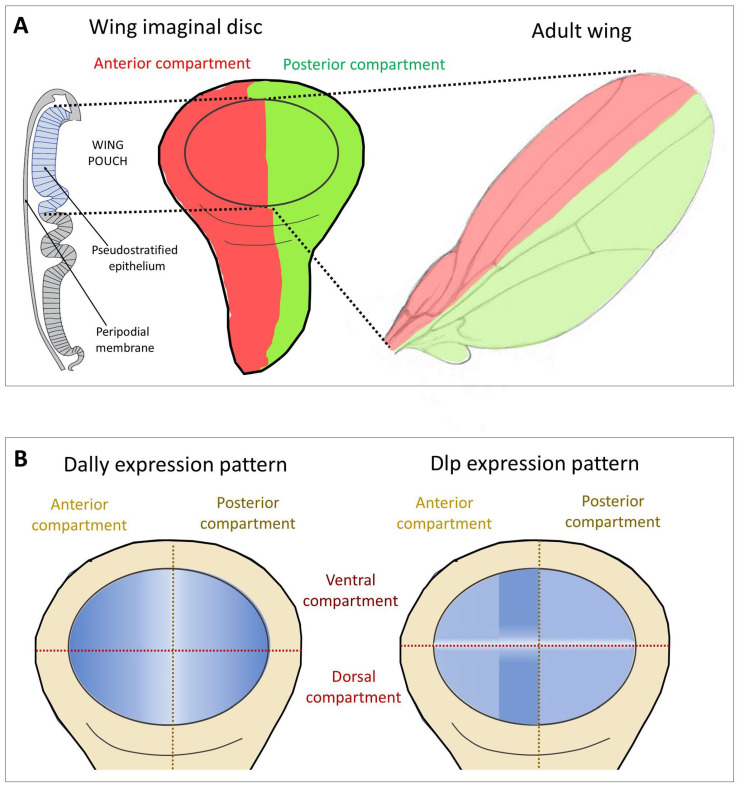
Wing imaginal disc as a model to study the function of Glps in Hh signalling. (**A**) The imaginal wing disc consists of a sheet of epithelial cells that form a sac-like fold of epithelium in fly larvae, called the wing pouch (marked in blue in the schematic representation of the polarised epithelium of the wing disc in a transverse section shown on the left), overlain by a fluid-filled closed compartment called the peripodial space (shown in white). Hh is produced throughout the posterior (P) compartment of the epithelial layer of the disc (green) and moves into the anterior (A) compartment to bind the Ptc receptor on the same epithelial layer (red). During this movement, Hh is thought to form a gradient of decreasing concentration with increasing distance from its source. Note that Hh movement must be confined to the epithelial layer to prevent morphogen loss into the overlying peripodial space, effectively ruling out free Hh diffusion as the underlying transport mode. Instead, Hh movement is thought to be confined to the epithelial layer by Glps. (**B**) Expression patterns of the Glps Dally and Dlp in the wing imaginal disc. Note that Dally shows reduced expression levels in the central area of the A-P boundary. Dlp shows higher expression levels in the A compartment adjacent to the P compartment and a marked decrease along the dorsoventral (D-V) axis.

Glp expression during *Drosophila* development is highly dynamic and tissue-specific [44]. The role of Glps in cell signalling has been largely determined in the developing wing disc, which later gives rise to the adult fly wing (Figure 2A). Dally and Dlp show a generalised expression, a positive modulation of Dally and Dlp levels has been observed in two regions of the wing pouch (Figure 2B). Dally expression is down-regulated in cells near the anterior-posterior (A-P) compartment boundary [45]. In contrast, Dlp protein is distributed in most disc cells except for a region centred on the D-V boundary (white stripe in Figure 2B). Importantly, these regions correspond to areas where several signalling factors and their receptors are expressed, suggesting that Dally and Dlp influence the formation of the morphogenetic gradients of Decapentaplegic (Dpp, BMP in vertebrates), Wg (the *Drosophila* Wnt) and Hh, in addition to the FGF and Jak/Stat pathways in the wing disc.

### 3.1. Dpp

Dpp is expressed at the A-P compartment boundary in the wing disc [46,47], and is induced by the Hh signalling pathway. Dally appears to play a more important role than Dlp in establishing the Dpp gradient [48] through the interaction of Dally core protein with Dpp [48]. Dally binds and stabilises Dpp on the cell surface and has been shown to be involved in both signalling (acting as a co-receptor) and ligand spreading [49,50]. In addition, Dally delays the degradation of the Dpp receptor complex, thereby potentiating Dpp signalling [50]. Recently, it has been shown that Dally HS chains have the function of stabilizing Dpp on the cell surface by antagonizing Dpp internalisation through its receptor Tkv [51]. The secreted protein Pentagon (Pent, also known as Magu) has been described as also being required for Glp activity in the formation of long-range Dpp gradients [4,51,52,53]. Pent has been shown to interact with the HS chains of Dally [4,52], raising the possibility that the interaction of the HS chains of Dally with Pent is critical for Dpp stability and thus for scaling the Dpp gradient. In this context, tunable levels of Pent regulate Glps to maintain an optimal balance between delayed receptor degradation and functional inhibition of the ligand [4]. 

### 3.2. Wg

Glps are thought to primarily help establish the Wg gradient at the D/V axis of the wing disc where they are expressed [45,54,55,56,57,58,59]. Here, Glps play a cell-autonomous role in receiving Wg signals and both Glps help to stabilise Wg at the cell surface [45]. As for Dpp, it has been proposed that the role of Dlp in Wg transport across cells is based on its ability to “transfer” Wg from one Dlp molecule to the next [60,61]. In this case, the core Dlp protein can interact with Wg (described in more detail in the next section), and the HS chains enhance this interaction [60]. To form the gradient, the secreted Wg antagonist Notum [62] and Dlp work together to restrict Wg signalling. This function of Notum can be explained by the conversion of Dlp from a membrane-tethered coreceptor to a secreted antagonist [57,62]. Another proposed mechanism underlying Notum’s suppressive activity on Wg signalling is ligand deacylation, a posttranslational modification that renders Wg inactive [63]. In this mechanism, the role of Glps has been suggested to help the carboxylesterase Notum to co-localize with Wg ligands at the cell surface. In the absence of Notum, Dlp shows a biphasic role in the activity of Wg morphogen in the wing disc: Dlp represses short-range Wg signalling while simultaneously activating long-range signalling [57,58,60,62]. The transition from signalling activator to repressor is determined by the relative expression levels of Dlp and the Wg receptor Frizzled2 (Fz2) [57,60]; thus, the ratios of Wg, Fz2, and Dlp are essential.

Another important property of Wg and Wnt proteins that leads to their association with the Glp core protein is their hydrophobicity as a result of their palmitoylation. Structural analysis has shown that in the presence of palmitoylated peptides, Glps change their conformation to create a hydrophobic space. In this way, Dlp family Glps can accommodate the Wnt/Wg lipid and protect it from the aqueous environment, thus acting as a reservoir from which Wnt/Wg proteins can be delivered to signalling receptors. Glp6 and Glp4, which form the Glp subfamily with the highest homology to Dlp, are also able to bind to the palmitate moiety of Wnt. In contrast, Dally and the mammalian Glps with the highest homology to Dally (Glp3 and Glp5) are unable to interact with palmitate moieties attached to a modified peptide. Based on these observations, it has been proposed that Dlp acts by sequestering the hydrophobic palmitate during Wg spreading and reception in the aqueous extracellular environment [64]. 

## 4. Glp Functions in the Hh Signalling Pathway in *Drosophila*

Glps are also critically involved in the formation of Hh gradients in *Drosophila* wing imaginal discs. Glp regulation of Hh can be stimulatory or inhibitory and can also occur at the level of signal reception. All of these regulations occur on physiologically relevant Hh proteins that have undergone double lipidation during their biosynthesis, but not on artificially produced monolipidated or unmodified Hh. In the following sections, we will focus on a detailed description of the function of Glps in Hh gradient formation in *Drosophila*.

### 4.1. Functions of Glps in Cells That Secrete Hh

In larval imaginal wing discs, Hh is produced and secreted by posterior (P) compartment cells and transported to the receiving anterior (A) compartment cells. Hh distributes across 8–10 rows of cells in the A compartment, forming a concentration gradient that decreases with distance from the A/P compartment boundary. Within the gradient field, Hh activates its targets in a concentration-dependent manner: Engrailed (En) and the canonical receptor Patched (Ptc) represent the high threshold targets close to the A/P boundary, Dpp and Collier (Col, also known as Knot (Kn)) are intermediate threshold targets, and Cubitus interruptus (Ci) and Iroquois (Iro) are low threshold targets [65,66].

A defining characteristic of the Hh protein family is their lipophilicity, which gives them a high affinity for membranes. Hh is initially produced as a 45 kDa precursor molecule that undergoes two post-translational lipid modifications: one by cholesterol, which is covalently attached to the C-terminus after processing and truncation of the Hh precursor into the 19 kDa-signalling protein [7], and the other by palmitic acid at the N-terminus of the signalling protein [8]. Both lipids tightly associate Hh with the plasma membrane of the producing cell, thereby limiting its ability to move freely through the extracellular medium [67]. While it has been shown that engineered Hh variants lacking the cholesterol modification (but still undergoing N-palmitoylation) have reduced signalling capabilities in vertebrate models [7,9,68], only minor effects on signalling have been reported in the *Drosophila* wing disc [69,70,71]. In contrast, the absence of N-terminal acylation significantly reduces signalling strength in both *Drosophila* and vertebrates [68,70,72,73,74,75]. Two main concepts have emerged in the past to explain these findings mechanistically. The first concept postulates that both lipids play an important role in determining the signal strength at the level of Ptc. In support of this concept, recent structural studies have suggested a functional role of one [76] or both lipids [77,78] in Ptc binding and signalling. Another concept postulates that both lipids play an important role in the spatio-temporally regulated release and transport of Hh from the producing cell surface to the anterior receiving cells. Indeed, spatio-temporally regulated Hh release mediated by the well-established multi-span transmembrane protein Dispatched (Disp) depends on the presence of both Hh lipids, whereas the artificial monolipidated protein variants described above undergo unregulated solubilisation independent of Disp function [69,79]. The important implication of the latter concept therefore is that the choice between regulated Hh secretion and release from the plasma membrane of producing cells, or the decision to remain membrane-associated, may determine the mode by which Hh is transported through the gradient field, which in turn may determine gradient formation and signalling strength within the morphogenetic field.

**Figure 3:**
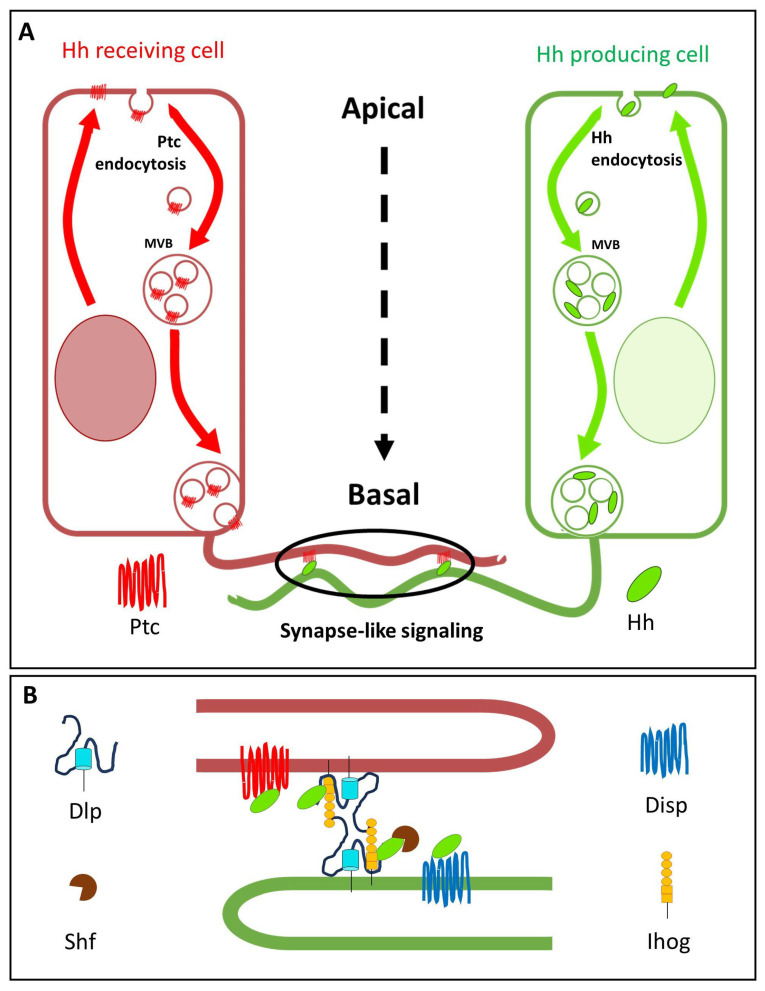
Schematic of a possible mechanism of presentation and reception of Hh. (**A**) Model showing direct recycling of Hh and Ptc to the basal surface of the *Drosophila* wing disc epithelium. Hh (green) and Ptc (red) undergo vesicular trafficking from the apical to the basal surface of their respective expressing cells by endocytosis and multivesicular body (MVB) formation (arrows indicate the direction of vesicular trafficking). At the basal surface, thin extensions of the plasma membrane called cytonemes mediate the transfer of Hh from Hh-producing cells in the posterior (P) compartment to Ptc-expressing, Hh-receiving cells in the anterior (A) compartment [12,80,81]. Reception occurs at specific contact points on cytonemes, similar to synaptic boutons [85,86]. (**B**) Schematic of the synaptic process involved in Hh signalling. Hh is released to be received by Ptc at contact sites on cytonemes. Hh release from producer cells may involve Dispatched (Disp), Ihog, the Dally-like proteoglycan (Dlp), and the diffusible protein Shf. In receiving cells, Dlp and Ihog proteins are involved in the co-reception of Hh.

Another crucial question regarding the formation of gradients on epithelia such as the wing disc is whether they form apically or basolaterally. Two opposing models have been proposed to explain how Hh is distributed in the wing disc epithelium. One model is based on the observation that lipid-modified Hh, although first localised to both the apical and basolateral membranes of Hh-producing cells, moves from the apical to the basolateral membranes via a vesicle-based intracellular trafficking pathway and is predominantly released from the basolateral pool. Similar trafficking of the Hh receptor Ptc from apical to basolateral plasma membranes takes place in the Hh receiving cells of the A compartment [80] (Figure 3A). Therefore, it has been suggested that the long-range Hh gradient is formed basolaterally, while the apical pool signals juxtacrinely at the maximum level of reception [3,12,81]. Another model [82] proposes that Hh is released apically from P compartment cells, moves into the luminal space, and is taken up apically by A compartment cells to form the long-range Hh gradient. In this apical Hh gradient, Hh is internalised in a dynamin-dependent manner, but it is then recycled back to the apical surface of A cells [83,84]. At the same time, a separate process releases and receives Hh basolaterally for the activation of high-threshold targets at short distances.

### 4.2. Glps Are Required for Hh Reception in A Compartment Wing Disc Cells

**Figure 4:**
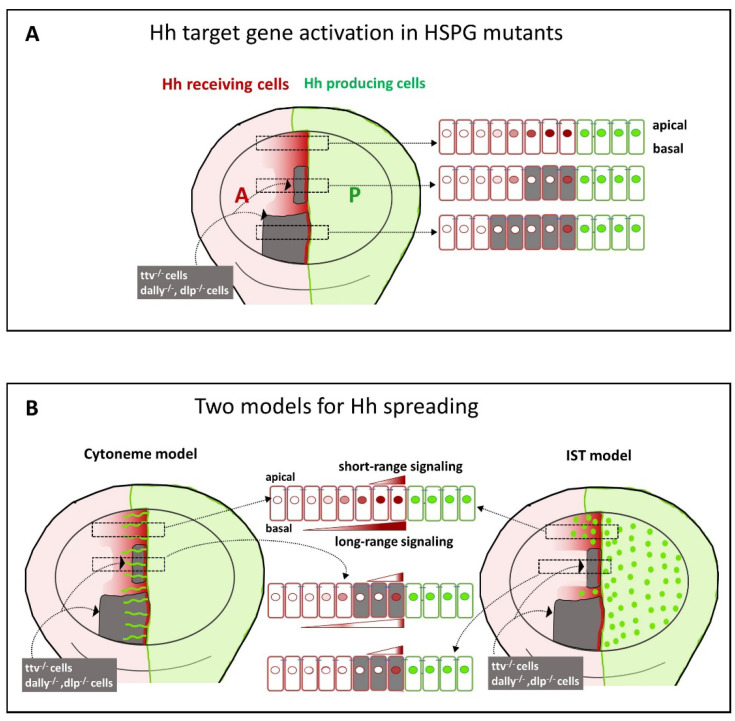
Effects of HS or Glp mutagenesis on Hh signalling in the wing disc and proposed mechanisms to explain these effects. (**A**) The wing imaginal disc, which later develops into the *Drosophila* wing, is divided into two compartments: the posterior (P) compartment, where Hh is produced (green), and the anterior (A) compartment, where Hh signals and induces the expression of target genes (red dots). Mutations in Glps, the HS biosynthetic enzymes of the EXT family, or in genes that determine the degree of N- and O-sulfation (such as the *Drosophila* gene *sulphateless,* clonal tissue is shown in grey) disrupt the formation of the Hh-signalling gradient in the A compartment (shown in red) [82,87,89]. Under these conditions, only clone cells directly adjacent to the P compartment are able to activate Hh signalling targets [17,70,87,89]. However, when the mutant clones are narrow, it has been shown that the low-threshold Hh-signalling targets can be activated beyond the anterior clone boundary, but not in all published experiments. (**B**) Two models have been proposed to explain how Hh spreads to the A compartment: the cytoneme model and the intersegmental transfer (IST) model. The cytoneme model suggests that cytonemes can traverse mutant territories and deliver Hh to distant cells as long as the mutant territories are small [17,85]. The IST model postulates direct morphogen transfer (green dots) from one Glp–HS chain to the next in the gradient field. In this model, lack of HSPG expression in small or large clones disrupts the formation of the Hh-signalling gradient in the A compartment (red) but still allows target activation in cells immediately adjacent to posterior Hh-producing cells.

The requirement of the HSPG/Glps for Hh reception was first demonstrated in mosaic analyses in the wing disc. These studies showed that lipid-modified Hh was only able to activate its targets in the first row of cells at the border of *ttv^-/-^* clones adjacent to the A/P compartment (Figure 4A). More anterior HS-deficient *ttv^-/-^* cells failed to respond to the Hh signal, reflecting problems with Hh stabilisation and/or dispersion through the clone [37], [38,87,88,89]. However, when the *ttv^-/-^* clones adjacent to the A/P boundary are small, activation of low threshold Hh target genes in wild-type (HSPG-expressing) cells anterior to these clones can still be observed in some [17,70,87,89] (Figure 4A), but not in all studies [90] (Figure 4B). Similarly, when both Dally, Dlp, or the EXT polymerase Botv are removed, a thin stripe of Ptc and Ci expression anterior to the A/P compartment boundary is still maintained [87,91,92,93] (Figure 4B). One explanation for this juxtracrine activation, as consistently observed within HSPG mutant clones, could be that the HS chains of Dlp and/or Dally in the wild-type P compartment cells function non-cell autonomously to support Hh signalling in the adjacent anterior mutant cells [60,94]. Note that the observed activation of juxtacrine signalling in HSPG-deficient cells does not necessarily indicate that Ptc in receiving cells requires a direct HS contribution for its receptor function, but that HS may also act to simply deliver the morphogen to the Ptc receptor on the surface of the receiving cells. 

The requirement of HSPGs for full activation of the Hh receptor and signalling pathway activation has also been demonstrated in ectopic Hh clones induced in the A compartment of the wing disc, in which the *ttv* gene required for HS biosynthesis has been eliminated [70]. However, there have also been reports of HS-independent activities due to Hh binding to the Glp protein core, although the full functionality of Glps depends on the HS side chains. Nevertheless, the Dlp core protein has been shown to be able to restore Hh signalling in a cell-autonomous manner in *dlp* mutant embryos, based on restored cuticle patterning following *prd*-Gal4-driven expression of Dlp without HS chains (Dlp(–HS)). The activity of HS-unmodified Glp core protein on Hh signalling has also been demonstrated in *Drosophila* cell lines. It was concluded that the core protein is essential for Dlp activity in Hh signalling, while the attached GAG chains confer additional non-cell-autonomous activity to Dlp [92,95]. The Dlp core protein has also been shown to interact directly with Hh [23], as similar levels of Hh accumulate on the surface of cells transfected with Dlp(–HS)-GFP or Dlp-GFP, and Hh co-immunoprecipitates with Dlp-GFP or Dlp(–HS)-GFP when co-expressed in S2 cells [92]. Finally, the C-terminal domain of Dlp, which links it to the GPI anchor and to the cell surface, is essential for its function in Hh signalling. However, the specific activity of the GPI anchor may not be necessary for Dlp’s role in Hh signalling, as the GPI anchor can be replaced with the transmembrane sequence of CD2 without affecting its function [92]. 

In contrast to the function of Dlp, the role of Dally in Hh reception may be primarily to concentrate Hh in the ECM. When *dally^-/-^* clones are present in the field of Hh-receiving cells, both Hh levels and the activation of Hh target genes are reduced [3]. Therefore, Dally is required to maintain Hh levels in the ECM of both the field of receiving cells [90,96] and the field of producing cells [3,93]. Again, Dally may not be a part of the Hh receptor complex on the receiving cell.

### 4.3. Glp Interaction with Components of the Hh Secretion and Receptor Complexes 

Several components of the Hh-signalling pathway have been shown to be specific for regulated Hh release from the producing cells (Figure 3B): the multi-span transmembrane protein Disp, the adhesion molecules, and Hh coreceptors Interference Hedgehog (Ihog) and Brother of Ihog (Boi) [97,98], the Glp Dlp and the soluble protein Shf. In addition, in Hh receiving cells, Hh binds to its receptor complex formed by its canonical receptor Ptc [99,100,101,102], the co-receptors Ihog, Boi [97,98] and Dlp [82,95,103,104]. We will now describe the known interactions of Glps with the above-mentioned components of the Hh-signalling pathway involved in Hh release and reception. 

### 4.4. Glps Interact with Disp

An important component of Hh signalling required for the release of cholesterol-modified Hh from producing cells is Disp; a 12-span transmembrane protein with a sterol sensing domain (SSD) similar to that of Ptc and proteins involved in sterol homeostasis and transport such as Niemann-Pick disease, type C1 (NPC1) and Sterol regulatory element binding protein cleavage activating protein (SCAP) [105,106]. Consistent with the presence of SSDs in these proteins, Ptc has recently been shown to transport free membrane cholesterol from the inner plasma membrane leaflet to an unknown acceptor [107], and Disp also depletes the plasma membrane of free cholesterol [105], but can also transfer the cholesteroylated C-terminal Hh peptide to soluble acceptors of the high-density lipoprotein (HDL) family [108]. These findings support previous observations of lipoprotein-mediated Hh transport in *Drosophila* and possibly also in vertebrates [10,11]. Although Disp has a generalised expression in the wing disc, which may reflect its function as a free cholesterol exporter, Disp activity is also specifically required in Hh-producing cells. Consistent with its function in extracting the cholesteroylated Hh C-terminus from the plasma membrane, in the absence of Disp, Hh is retained in P cells and its access to A cells is severely restricted [69,109]. Of note, the *disp^-/-^* embryonic phenotype mimics that produced by the absence of the Glp Dlp [81,103,104,110], and both *disp^-/-^* clones and *dlp^-/-^* clones show increased Hh levels in the Hh-producing P compartment cells in the wing disc. Furthermore, Disp overexpression increases Dlp levels at the basolateral side of the epithelium, suggesting a Disp-Dlp interaction in the process of Hh release from producing cells [81]. Given the possible role of Disp in the vesicular trafficking of Hh [81,83], Disp may also regulate the recycling of Dlp from the apical to the basolateral plasma membrane. The Disp–Dlp interaction was confirmed by immunoprecipitation studies [81].

### 4.5. Glps Interact with Ptc

As mentioned above, Dlp is required for Hh signalling in a cell-autonomous manner. It is known to act upstream or at the level of the canonical Hh receptor Ptc [104] in cultured cells, during embryonic development [96,103,104] and in wing imaginal discs [45,90,95,104]. Similar Hh-stimulatory activity has been reported for Gpc5 and Gpc6 in mammalian cells [111,112]. Gpc5 and Gpc6 interact with both Hh and Ptc and stimulate the interaction between Hh and Ptc [112,113]. The increased interaction of Hh with Ptc promoted by Dlp could further reduce the availability of unbound Hh in the matrix, thereby reducing the signalling range [92]. Dlp can also co-precipitate with Ptc-GFP, suggesting that Dlp may facilitate or bridge the interaction between Ptc and Hh [92]. Since Dlp interacts with Disp for Hh release and with Ptc for Hh reception, Dlp could act as a relay for the transfer of lipid-modified Hh from the presenting to the receiving cells. Indeed, it has been proposed that Dlp acts as a lipid relay for the transport of lipid-modified Wg [64].

### 4.6. Glps Interact with Ihog and Boi

The two Hh coreceptors Ihog and Boi are type I transmembrane proteins, each with four immunoglobulin (Ig) and two fibronectin type III (FNIII) extracellular domains and an intracellular domain [98]. One of the FN domains of Ihog (FN1 domain), which is required to maintain Hh levels, also interacts with Glps [48,114]. Furthermore, Dally and Dlp are specifically required for the proper stabilisation of Ihog in plasma membranes, as Ihog levels decrease in cells lacking both Dally and Dlp (*dlp^-/-^ dally^-/-^*), while Boi levels remain unaffected [48]. The stabilisation of Ihog appears to be mediated by the Glp HS chains, as evidenced by reduced Ihog levels at plasma membranes in *ttv^-/-^ btv^-/-^* clones. Interestingly, the Dlp core does not immunoprecipitate Ihog [23], suggesting that the interaction may occur through the HS chains. Reception of Hh is completely abolished when the Hh coreceptors and adhesion molecules, Ihog and Boi, are simultaneously absent in the receiving cells [115]. Although their function in Hh gradient formation in the wing disc was thought to be redundant, the absence of each gene affects gradient formation in a specific way: Ihog is required for the long-range gradient, whereas Boi is required for the short-range gradient [48]. In addition to their roles in Hh reception, Ihog and Boi are separately required to maintain normal Hh levels in Hh-producing cells through their interactions with Glps [3,92]. 

### 4.7. Glps Interact with Shf

Shf, a diffusible component of the extracellular matrix in *Drosophila*, is homologous to the vertebrate Wnt inhibitory factor (Wif-1). Both Shf and Wif-1 proteins have a Wif box specific for Hh in *Drosophila* and for Wnt in vertebrates, and five EGF repeats. Shf appears to interact with the HS chains of Glps, since Shf levels in the extracellular matrix decrease when HS chains are absent in *ttv^-/-^* and *sotv^-/-^* mutant clones [15]. In the absence of Shf, the Hh gradient fails to form, probably because of a reduction in extracellular Hh levels, leading to a decrease in the expression of Hh target genes [14,15]. Interestingly, overexpression of Dally can still increase Hh levels in *shf* mutants, suggesting that Shf and Dally have complementary functions [3,93]. One possibility is that Shf helps to release Hh from its binding to Dally during the reception process. This hypothesis is supported by the observation that overexpression of Shf rescues the effects of overexpressed Dally in the P compartment, which otherwise hijacks Hh and thereby restricts Hh signalling [3]. An interaction between Shf and Dally [3] has been confirmed by co-immunoprecipitation, while no interaction between Shf and Dlp has been found to date. The Dally interaction appears to occur through the EGF repeats of Shf since mutants in one of these repeats (*shf^2^* mutant allele) prevent Shf accumulation at the cell surface when Dally is overexpressed [110]. Similarly, the EGF-like domains of Wif-1 have been shown to bind HS in vitro [116]. Finally, Dally appears to stabilise Shf protein in the extracellular space: when Dally is removed or overexpressed, Shf levels decrease or increase accordingly [3,110,117]. 

## 5. Role of Glps in Hh Transport and Gradient Formation 

As described above, the graded distribution of morphogens must be tightly regulated to ensure proper tissue formation. Since Hh molecules are highly modified by lipids that attach Hh to the plasma membrane, specific mechanisms are required to release and distribute Hh in the morphogenetic field and to regulate its signalling capacity. Depending on the mode of Hh release, two main transport mechanisms are expected to regulate the distribution of the extracellular Hh ligand, and both mechanisms require Glps to play an important regulatory role: First, Glp-modulated transport of micellar, exosome-associated, LPP-associated or proteolytically processed Hh by some form of extracellular direct relay to target cells and, second, Glp-modulated, cytoneme-mediated cell–cell communication (Figure 4B). Since both transport mechanisms depend on extracellular HSPG expression in the gradient field, the above findings on the role of HSPGs in Hh transport can be explained by both models, as described in the following sections.

### 5.1. Direct Hh Hand-Over between Glp HS Chains in the Morphogenetic Field

**Figure 5:**
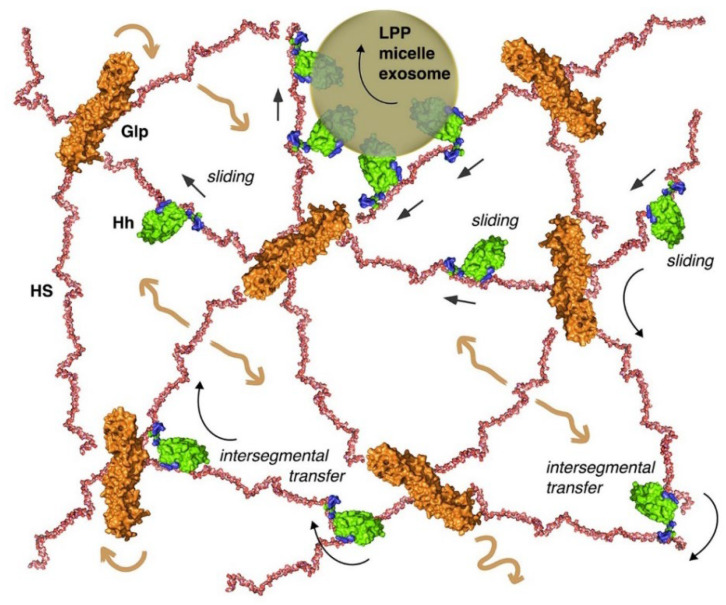
Proposed Hh relay by the IST or “monkey bar” mechanism. In the monkey bar (or intersegmental transfer (IST)) mechanism, two HS-binding domains on the Hh protein interact simultaneously with one or two HS chains. In the first situation, the protein can move along the sugar chain (sliding) and in the second situation, it can switch directly between them without a diffusible intermediate (intersegmental transfer). The process is initiated by flexible N-terminal tail interactions with the new acceptor chain, followed by Hh switching through an intermediate in which the second binding site is retained on the original HS chain. This “monkey bar” mechanism avoids intermittent steps of free protein diffusion and protein loss from the HS chain. We note that the same mechanism could potentially be used by Hhs associated with lipid carriers such as lipoprotein particles [10] and exosomes [13], or after their association in micelles [9]. Shown are the pdb structures 3irl (HS), 4ad7 (Glp), and 3m1n (Shh). The structures are not to scale. Brown arrows indicate the dynamic movement of Glp HSPGs on the cell surface and black arrows indicate electrostatically restricted Hh movement on and between the HS chains.

The model of direct morphogen transfer from one Glp–HS chain to the next may apply to Hh associated with Shf, to delipidated Hh released as a consequence of double processing of the two terminal lipidated Hh peptides, or to Hh attached to LPPs or other lipid carriers (Figure 5). One mechanism by which all these forms of Hh could be distributed on developing epithelial surfaces is through electrostatic interactions between the negatively charged Glp–HS sugar–sulfate chains and two basic HS-binding sites on the Hh morphogen [118,119]. These direct electrostatic interactions guide and constrict extracellular Hh transport to the HS-rich epithelial surface to maximise the efficiency with which Hh searches for its receptor, a process termed “sliding” (Figure 5). In addition to the possibility of both Hh binding sites interacting with the same extracellular HS chain, each binding site may also interact directly with different neighbouring chains to switch directly between them and move in the gradient field, a process termed “intersegmental transfer (IST)” (Figure 5). This extracellular transport mode is strikingly similar to the mode used by DNA polymerases, transcription factors, nucleases, and other DNA-binding proteins in the nucleus [120]. Similar to HS-binding proteins that interact with the sugar–sulphate HS chain, DNA-binding proteins associate with the negatively charged sugar–phosphate DNA backbone through non-specific, long-lived electrostatic interactions [121,122]. These electrostatic attractions prevent the protein from diffusing away from DNA but allow it to move by sliding along the axis of the double helix to find its targets more quickly. During this process, one binding site occasionally remains associated with one strand of DNA while the other binding site engages in target search as a prerequisite for inter-DNA transfer or intersegmental transfer [123]. Importantly, the intersegmental transfer is characterised by direct protein transfer between negatively charged sugar backbones. Protein movement by intersegmental transfer can be illustrated by children playing “monkey bars”, moving from one bar to the next, always holding one bar—and occasionally both bars—to prevent falling. Intersegmental protein transfer therefore differs from previously proposed mechanisms postulated for proteins with a single binding site. Such proteins move by repeated cycles of sugar–polymer binding, unbinding, passive diffusion, and sugar–polymer rebinding of the protein, called “hindered” or “restricted” diffusion.

Support for extracellular intersegmental protein transport along HS chains came from site-directed mutagenesis of one of the two established HS binding sites of Hh [16,124] and Shh [124,125]. This strategy converted both proteins into classic on/off binders with only one fully functional HS binding site, converting intersegmental transfer between HS into restricted protein diffusion. When expressed under the control of the endogenous Hh promotor in *Drosophila* eye and wing discs that have also been rendered null for endogenous Hh function [126], the mutant Hh protein caused the selective loss of eye and wing tissues known to require HSPGs for Hh long-range spreading. In contrast, other fly tissues from the same disc that depend on short-range Hh signalling developed normally, demonstrating that the mutant protein was selectively impaired in its ability to move between multiple HS chains for long-range relay, but less so in cell-to-cell signalling and in its ability to bind to its receptor on target cells in vivo [118] that could also be achieved by the solubilised diffusing ligand. Importantly, *en*-Gal4/UAS-controlled overexpression of the same proteins with only one fully functional HS binding site in the posterior wing disc compartment induced ectopic signalling at the peripodial membrane, which overlies the actual wing disc and is separated from this epithelium by a narrow, fluid-filled, closed compartment called the peripodial space (Figure 2). This observation confirmed that the Hh protein, which is normally restricted to the epithelial apical surface of the disc epithelium (possibly by the process of intersegmental transfer that avoids intermittent free diffusion steps) is transformed into a soluble, freely diffusing molecule that is able to cross the peripodial space and reach the peripodial membrane. This observation also highlights the importance of direct Hh handover from one HS chain to the next via its two HS binding sites for robust gradient formation; it also suggests that Hh handover could occur at the apical side of the wing disc to form long-range gradients, as previously proposed [71,82,127,128]. Alternatively, the increased diffusion of Hh mutated in one of the HSPG binding sites, which move freely on the apical epithelial surface, may have hindered Hh recycling to the basolateral side to form the basolateral gradient. This effect is similar to that produced when a non-membrane anchored form of Dally (Dally secretable) is expressed in the Hh-producing cells. As mentioned above, this form of Dally prevents the retention of Hh at the apical membrane and therefore interrupts its recycling towards the basal side of the epithelium [3] to form a basolateral long-range gradient [12,17,48,80,81,85,86,129].

**Figure 6:**
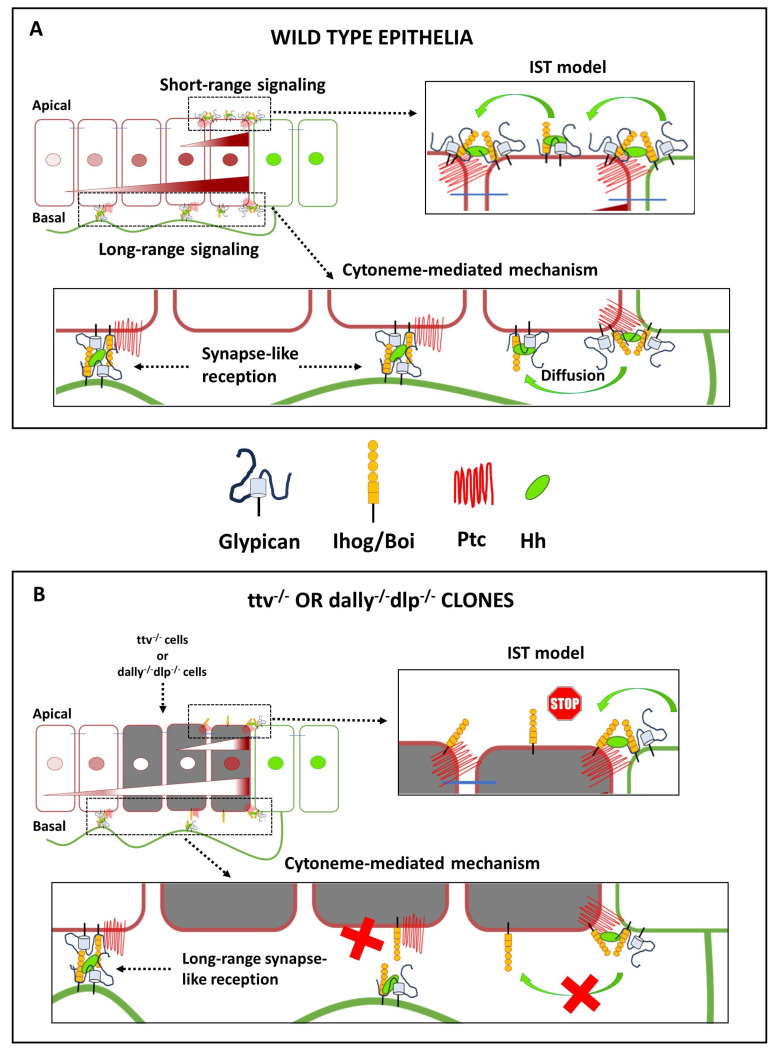
Possible roles of HSPGs in the two models of Hh signaling. (**A**) During Hh trafficking in wild-type epithelia, Hh protein is exchanged between Hh-producing and Hh-receiving cells in a process that requires Glp HSPGs. One possibility is that Glp-associated Hh moves directly on the apical cell surface via the HS chains of the Glps to form a signalling gradient (short-range but possibly also long-range). In the cytoneme-signalling model in wild-type epithelia, Hh is exchanged at basal cytoneme contact sites in a synapse-like process. In this exchange process, Glps play a key role in facilitating both cytoneme contact and Hh reception. (**B**) Hh transport in Glp-HSPG-deficient clones (*ttv^-/-^*, or *dally^-/-^ dlp^-/-^*), signalling occurs only in the first row of Hh-receiving cells of the A compartment. This is presumably because Ptc can contact Hh exposed by the Hh-producing cells, with the HSPGs in the P compartment possibly rescuing the first row of cells touching the A/P boundary in a non-autonomous manner. In the clone interior, however, Hh-loaded cytonemes protruding from Hh-producing cells are unable to contact the Glp-deficient Hh-receiving cells, preventing the activation of Hh-signalling targets. However, Hh-loaded cytonemes can pass through these Glp-deficient areas and contact wild-type cells anterior to the clone, allowing activation of low Hh-signalling targets far from the A/P compartment boundary. Alternatively, the IST model proposes that the lack of HS acceptors for mobile Hh in the HSPG-deficient clone inhibits Hh transport at the cell surface and traps Hh at the clone boundary.

To provide a mechanistic explanation for the in vivo findings, a technique called quartz crystal microbalance with dissipation monitoring (QCM-D) was used. The strength of the QCM technology is that it allows real-time detection of Hh interactions with heparin–the most highly sulphated form of HS–on fluid-supported lipid bilayers at the sensor surface. A second advantage of this technology is that heparin is coupled to supported lipid bilayers and can therefore freely rotate and move laterally on the sensor to mimic HS attached to GPI-linked Glps on the cell surface (Figure 1). The third advantage is that QCM-D measures an additional parameter, the change in energy dissipation *D*, which indicates changes in the stiffness of the supported lipid/heparin layer. This additional parameter revealed that Shh not only binds heparin but also effectively cross-links the heparin chains on the sensor, consistent with the presence of two functional Shh binding sites [125]. In contrast, the ability of the Shh mutant to cross-link heparin, e.g., to bind two sugar chains at the same time, was greatly reduced when mutated to retain only one fully functional HS binding site in these proteins. When soluble heparin was added to the wash buffer as a potential acceptor, it was observed that soluble heparin rapidly eluted most of the Shh from the QCM-D sensor surface, but not the mutants. This observation showed that both functional Shh binding sites bind two HS/heparin chains simultaneously [125] and transiently as an intermediate during the repeated Shh “monkey bar” movement from one sugar chain to the next, a property that may explain their in vivo spread [90] (Figure 6A, IST model is shown on the right). Notably, equal amounts of selectively desulphated soluble heparins did not elute Shh from the sensor surface, demonstrating that Shh moves rapidly and directly between sugar-sulphate chains of equal total charge, but does not switch from higher to lower sulphated chains or in the absence of acceptor HS. This may explain the fraction of in vivo studies showing that Hh cannot spread across clones deficient in the HSPG biosynthetic genes *sfl*, *ttv*, and *dally/dlp*, and why Hh accumulates at the clone boundary [90]. This latter observation also supports the view that Hh is not relayed by restricted diffusion since the absence of HSPG in the clone would facilitate anterior Hh spreading rather than slowing it down.

### 5.2. Glps and Cytonemes Work together to Distribute the Hh Ligand

In contrast to the model proposing that Hh dispersal is facilitated by direct Hh hand-over between Glp HS chains, an alternative model suggests that cytonemes mediate the long-distance distribution and reception of Hh (Figure 4B and Figure 6A). These structures have been observed in various *Drosophila* and vertebrate systems and in several signalling pathways [130,131,132,133]. In the context of Hh signalling, it has been observed that cytonemes of Hh-producing cells extend across the morphogenetic gradient. This has been observed in both wing discs and abdominal histoblast nests [17]. In vivo imaging of abdominal histoblast nests has shown that cytonemes extend and retract dynamically. The establishment of the Hh gradient has been observed to correlate with cytoneme formation in both space and time [17]. Furthermore, Hh has been shown to be associated with vesicles transported along cytonemes [12], providing a link between cytoneme formation and observations of Hh release and transport along with various lipid particles.

In addition, cytonemes originating from the Hh-receiving cells in the A compartment also appear to play a role in Hh reception and gradient formation. These cytonemes show similar dynamics to those originating from the Hh-producing cells [17,85,86]. Interestingly, cytonemes from both signal-producing and signal-receiving cells connect at specific sites along their length. This suggests that these contact sites may facilitate transmission and reception of the morphogen [85,86]; mathematical modelling based on these dynamic contact sites predicts the correct experimental Hh gradient [129].

Cytonemes are located at the basal side of the *Drosophila* epithelium, aligning with the plane where Hh and its receptor Ptc are positioned after being recycled from the apical to the basolateral plasma membrane [80,81]. Furthermore, most Hh-signalling components, including Dally, Dlp, Ihog, Ptc, and Hh, colocalise with the signal-receiving cytonemes [3,48,80,85]. In Hh-secreting cells, Disp, Shf, Ihog, Dally, and Dlp are also localised in cytonemes [3,17,81] (Figure 3B). Taken together, these findings support a model in which cytonemes serve as conduits for Hh morphogen movement at the basal plane of the epithelium.

In vertebrates, including mammals, cytonemes also play a crucial role in directing the long-range distribution of Sonic Hh during patterning [134,135]. Similar to *Drosophila*, this transport from the producing cells requires the involvement of the vertebrate Disp and BOC/CDON [136], which are homologous to Ihog and Boi [137], as well as Myosin 10 [136,138]. In a study using optimised in vivo imaging, it was shown that a complex array of filopodial extensions forms upon limb amputation in axolotls and that these extensions play a key role in re-establishing the Shh signalling gradient also during the regeneration process [138].

The importance of HSPGs in the navigation of cytonemes is demonstrated by the behaviour of the HSPG mutant clones adjacent to the A/P compartment boundary described above. In these clones, it has been shown that Hh is unable to cross the mutant territory and that Hh cannot signal to wild-type cells anterior to the HSPG-deficient clone [87,89]. A facilitated diffusion model was initially proposed to explain this effect [46], suggesting repeated cycles of HSPG attachment, detachment, free diffusion, and reattachment to keep the morphogen close to the cell surface. This mechanism differs from the IST mechanism described above, in which the morphogen is directly “handed off” from one HS chain to the next in the morphogenetic field. On the other hand, if cytonemes are involved in Hh transport, they may or may not be able to cross a mutant territory to deliver the signal across the clone. In this regard, it was shown that cytonemes stabilised by Ihog overexpression did not cross either *ttv^−/−^ botv^−/−^* [17] or *dlp^-/-^ dally^-/-^* [85] mutant clones, and consequently no Hh response could be detected anterior to these clones. However, when the clones were very narrow, Ihog-stabilised cytonemes crossed the mutant territory and Hh responses were detected in the anterior wild-type compartment [17] (Figure 6B). These results still suggest a requirement of Glps for cytoneme stabilisation and their specific role in the cytoneme interplay between Hh-producing and Hh-receiving cells. 

### 5.3. Glps in Cytoneme Stability, Dynamics and Guidance

A role for Glps in cytoneme formation has been described during communication between neighbouring epithelia in *Drosophila*: dorsal air sac (ASP) development depends on Dpp and FGF proteins produced by the wing imaginal disc and transported by cytonemes to the air sac primordium. Dpp-receiving ASP cytonemes require Dally (but not Dlp), whereas FGF-receiving ASP cytonemes navigate in the Dlp layer and require Dlp (but not Dally) for their formation [139]. Similarly, in the context of vertebrate Wnt signalling, Glp 4 mediates the delivery of Wnt ligands (Wnt5b and Wnt11f2) through cytonemes from Wnt-expressing endodermal cells to mesodermal and ectodermal cells [140].

The growth and dynamics of cytonemes require interaction with extracellular matrix components. Ihog plays a specific role in stabilising cytonemes, whereas Boi does not play a similar role [48]. The ability of Ihog to control cytoneme dynamics depends on its interaction with Glps. This Ihog–Glp interaction is facilitated by the two FNIII domains of Ihog [48,114]. Interestingly, these are the same domains that have been described to interact with Hh and Ptc [48,97,115,141].

While the requirement for HSPGs in cytoneme dynamics is established, it is not yet clear if they participate in the commitment of cytonemes to grow in a particular direction. In zebrafish, Glp 4 has been identified as playing a role in guiding cytonemes involved in Wnt signalling [140]. In *Drosophila*, experimental results have shown that cytonemes from a cell population with high levels of Ihog are stabilised and oriented towards another cell population with high levels of either Dally or Dlp expression and those from the latter towards the former [85]. Cytonemes originating from a region simultaneously overexpressing Ihog and Dally or Ihog and Dlp are not stabilised and their orientation cannot be ascertained [142]. These results suggest that the stabilisation of cytonemes may be due to molecular competition depending on the availability of Ihog and Glps. Based on these experimental results and taking into account the protein levels and distribution of Ihog, Dally, and Dlp in the wing imaginal disc a mathematical model was constructed to predict the guidance of cytonemes for Hh signalling [142]. This in silico model is able to simulate the orientation of the cytonemes under conditions of overexpression of Ihog and Dally or Ihog and Dlp as well as in wild-type discs.

## 6. Concluding Remarks

As discussed in this review, Glps play important roles in lipid-modified Hh signalling. In both Hh-producing and Hh-receiving cells, Dally and Dlp play critical roles: Dally plays a role in stabilising Hh in membranes by maintaining adequate Hh levels and preventing its free spread, and Dlp plays roles as a co-receptor in the cells that receive Hh and in the release of Hh from the cells that produce it. The co-receptor function depends on the Dlp core while the non-cell autonomous activity is provided by the GAG chains.

Glps interact not only with Hh but also with other essential components of the Hh pathway: (1) Dlp has a direct interaction with the transmembrane proteins Disp and Ptc. Dlp interaction with Disp facilitates the release of Hh from the producing cells, and Dlp interaction with Ptc facilitates the binding of Hh to its receptor. (2) Dally may also help to stabilise Hh at the plasma membranes through its interaction with Shf, a diffusible protein essential for the proper distribution of lipid-modified Hh. Both Glps interact with the Hh coreceptor Ihog and help to stabilise it at the plasma membrane.

In summary, Glps play a crucial role in the movement of Hh through the extracellular matrix and in long-distance cell–cell communication. Two models have been proposed on the role of Glp in long-range gradient formation: In the IST, or monkey bar model, the HS chains of Glps repeatedly transfer Hh directly from producing to receiving cells. Importantly, the degree of HS modification appears to control this movement, providing an explanation for the robustness of the Hh gradient and its ability to be scaled simultaneously (through the net charge differences between donor and acceptor HS chains) [118]. The model, therefore, proposes that Hh moves from HS chains with relatively low negative charge to more sulphated HS with an increased net negative charge, but not back, which would also give directionality to the movement. The cytoneme model also explains long-distance Hh signalling with considerable confidence [129]. Glps are also crucial in this model: first, in the stabilisation of cytonemes by Ihog [17,48,85,114]; second, in the guidance of these dynamic cellular structures [85,142] and third, in the establishment of contacts between cytonemes for the release and reception of Hh [80,85,86]. When interpreting the behaviour of the Hh gradient in a small region of HSPGs mutant clones, it is important to note that cytonemes can cross this region and signal anteriorly to these mutant cells. In contrast, in vitro and in vivo assays that have led to the “monkey bar” model of Hh transport should not result in signalling anterior to HSPG-deficient cells. In both models, within the cloned tissue, Glps appear to provide a sufficient ability to rescue juxtacrine or short-range signalling to allow Hh signalling to the one row of mutant cells adjacent to the clone boundary but fail to activate signalling in more distant cells within the clone (Figure 6).

Since different Hh gradients can form at the apical and basolateral sides of the wing disc epithelium, the role of Gpls in Hh movement may be twofold in these two models. Therefore, the “monkey bar” model and the cytoneme model may act at different subcellular sites but still work together to ensure efficient and precise delivery of Hh to the receiving cells. The “monkey bar” type of movement may also be at work in the movement of Hh along the cytoneme membrane. Going one step further, the mechanism of Hh exchange between Glps, mediated by their HS chains and the HS/heparin binding sites of Hh, could assist in the exchange of Hh from presenting to receiving cytonemes. Indeed, it is possible that in these synapse-like contacts between cytonemes, the exchange of Hh is mediated by a short-range intersegmental transfer that likely requires the function of Glps. In this exchange, Dlp could act as a conduit for the transfer of lipid-modified Hh from presenting to receiving cells.
